# Reduced Sensitivity to Between-Category Information but Preserved Categorical Perception of Lexical Tones in Tone Language Speakers With Congenital Amusia

**DOI:** 10.3389/fpsyg.2020.581410

**Published:** 2020-09-30

**Authors:** Fei Chen, Gang Peng

**Affiliations:** ^1^School of Foreign Languages, Hunan University, Changsha, China; ^2^Research Centre for Language, Cognition, and Neuroscience, Department of Chinese and Bilingual Studies, The Hong Kong Polytechnic University, Hong Kong, China; ^3^Chinese Academy of Sciences (CAS) Key Laboratory of Human-Machine Intelligence-Synergy Systems, Shenzhen Institutes of Advanced Technology, Chinese Academy of Sciences, Shenzhen, China

**Keywords:** amusia, P300, acoustic processing, phonological processing, categorical perception

## Abstract

Previous studies have shown that for congenital amusics, long-term tone language experience cannot compensate for lexical tone processing difficulties. However, it is still unknown whether such difficulties are merely caused by domain-transferred insensitivity in lower-level acoustic processing and/or by higher-level phonological processing of linguistic pitch as well. The current P300 study links and extends previous studies by uncovering the neurophysiological mechanisms underpinning lexical tone perception difficulties in Mandarin-speaking amusics. Both the behavioral index (*d′*) and P300 amplitude showed reduced within-category as well as between-category sensitivity among the Mandarin-speaking amusics regardless of the linguistic status of the signal. The results suggest that acoustic pitch processing difficulties in amusics are manifested profoundly and further persist into the higher-level phonological processing that involves the neural processing of different lexical tone categories. Our findings indicate that long-term tone language experience may not compensate for the reduced acoustic pitch processing in tone language speakers with amusia but rather may extend to the neural processing of the phonological information of lexical tones during the attentive stage. However, from both the behavioral and neural evidence, the peakedness scores of the *d′* and P300 amplitude were comparable between amusics and controls. It seems that the basic categorical perception (CP) pattern of native lexical tones is preserved in Mandarin-speaking amusics, indicating that they may have normal or near normal long-term categorical memory.

## Introduction

As two old and unique products of the human brain, speech and music are present across all cultures (Patel, 2008). Although they belong to different domains with different representations, an increasing number of neuroimaging studies point to a large neural overlap in responses to speech and musical stimuli (see Peretz et al., 2015 for a review), which implies a close relationship between musicality and speech-processing capacity. Pitch, an important psycho-acoustical attribute in both music and speech, is often at the center of researchers’ attention in studies on the mutual influence between speech and music. Accumulating evidence suggests that experience in one domain could be transferred to facilitate processing in the other: for example, a tone language background is often associated with better performance in musical pitch processing (e.g., Pfordresher and Brown, 2009; Bidelman et al., 2013; Peng et al., 2013). On the other hand, compared with non-musicians, musicians tend to be more skilled at detecting non-native lexical tone contrasts (e.g., Wong et al., 2007; Lee and Hung, 2008; Chandrasekaran et al., 2009) and even more sensitive to native linguistic tone categories (Tang et al., 2016). These studies imply a two-way transferability of pitch expertise across the domains of music and speech.

From another perspective, the existence of congenital amusia (simply amusia hereinafter), a lifelong neuro-genetic disorder primarily affecting fine-grained melodic pitch processing in the absence of brain injury (Peretz, 2001; Peretz et al., 2002), offers an ideal window to investigate the influence of impaired musical ability on lexical tone processing in speech. As indicated in various behavioral studies, impaired or reduced lexical tone perception skills are consistently reported among non-tonal language speaking amusics (Nguyen et al., 2009; Tillmann et al., 2011) and in tone language speaking amusics at the whole group level (Jiang et al., 2012; Wang and Peng, 2014; Zhang et al., 2017a) or in a subgroup called “tone agnosics” (Nan et al., 2010; Huang et al., 2015). On the basis of these behavioral findings, we can conclude that the pitch processing deficit in amusia tends to be domain-general regardless of language background. The processing deficit in musical melody may be transferred to pitch-based speech processing.

Although there is ample behavioral evidence of pitch impairment in amusia, the evidence regarding the neurophysiological bases that could account for the nature of amusics’ pitch processing deficits is not completely conclusive. The structural abnormalities in the right inferior frontal gyrus (IFG) of amusics and its reduced connectivity with the auditory cortex (Hyde et al., 2006, 2007; Albouy et al., 2013) were corroborated in a functional MRI (fMRI) study (Hyde et al., 2011), which demonstrated a functional deactivation in these brain networks of amusics. However, the auditory cortex (superior temporal gyrus, STG) of amusic individuals seems to function normally to pitch change (Hyde et al., 2011), although brain structural abnormalities have been detected in this core auditory area (Hyde et al., 2007; Mandell et al., 2007). Nevertheless, there is conflicting evidence implying an anomalous functioning of the auditory cortex of amusics (Albouy et al., 2013; Zhang et al., 2017b). The discrepancy regarding the functioning of the auditory cortex could be partly explained by the experimental tasks utilized in these functional neuroimaging studies. Specifically, the normal functional activation of auditory cortices was found when subjects were passively listening to pitch deviations without focal attention (Hyde et al., 2011). However, abnormal functional activation of auditory cortices was shown in both non-tonal language speakers (Albouy et al., 2013) and tone language speakers with amusia (Zhang et al., 2017b) when the focal attention was directed to the auditory stimuli. Consequently, attentional requirements are likely to play a key role in the neurophysiological manifestation of amusia.

Consistent with this claim, accumulating evidence from event-related potential (ERP) studies converges with the notion that pre-attentive implicit processing of pitch information might remain normal in amusics, whereas their explicit judgment or awareness of pitch perception might be compromised (Peretz et al., 2005, 2009; Moreau et al., 2013; Omigie et al., 2013; Zhang and Shao, 2018; but see Nan et al., 2016 for evidence showing impaired pre-attentive tone processing in a small proportion of Mandarin-speaking amusics called tone agnosics). For instance, the mismatch negativity (MMN), which is an early component reflecting an automatic cortical response to auditory changes without subjects’ focal attention (Näätänen, 2000), was found to remain intact in amusics even for small 25-cent musical pitch differences; instead, unlike the controls who also showed a prominent P3b in the attentive condition, no P3b response was shown in the amusics when they were asked to actively detect such small pitch deviations in music (Moreau et al., 2013). In the speech domain, for tone language speakers with amusia, a recently conducted ERP study (Zhang and Shao, 2018) examined how typical Cantonese tone stimuli were processed in both pre-attentive (MMN) and attentive (P300) conditions and showed that Cantonese-speaking amusics could perceive lexical tone changes normally pre-attentively but showed a deficiency in consciously detecting the same lexical tone differences at the later attentive level (Zhang and Shao, 2018). In short, the findings above suggest a neuro-dynamic mechanism of intact pre-attentive pitch processing but impaired active/attentive pitch processing among amusics in the domains of both music and speech.

A plethora of studies have demonstrated that long-term access to a tonal language environment may shape the neural architectures of pitch processing in native speakers (e.g., Gandour et al., 2004; Bidelman et al., 2011; Gu et al., 2013). So far, the neurophysiological mechanisms underlying amusics’ behavioral linguistic tone deficits remain unclear. This topic is also related to the question of whether and how long-term experience of a tonal language affects the neural bases of amusia. It is well known that in a tonal language, the pitch is exploited to distinguish phonological contrasts and word meanings at the syllable level. In stark contrast to non-tonal language speakers, Cantonese-speaking amusics tend to exhibit no functional abnormality in the right IFG. Instead, they mainly exhibit neural deficits in the right STG when processing lexical tones, which partly overlaps with neural activation of lexical tone processing (Zhang et al., 2017b), implying that language background might modulate the neural correlates of amusics’ brains. Similar to non-tonal language speakers with amusia, at different processing stages along the auditory pathway, the lexical processing deficits in tonal language speakers with amusia have also been demonstrated to occur at a later time window when consciously detecting the lexical tone changes at the attentive stage (Zhang and Shao, 2018). However, it is still unknown whether, during this time window (P300), such an attentive lexical tone processing deficit in tone language speakers with amusia is restricted to the domain-transferred insensitivity to the acoustic pitch differences or, alternatively, further extends to a reduced phonological processing of lexical tone categories.

As suggested by the functional hypothesis (van Lancker, 1980), the accurate and complete perception of lexical tones in native tone language speakers involves the processing of acoustic information as well as the phonological information carried by the tonal signal. Specifically, the acoustic information contains pitch features, such as pitch height and contour variations, while the phonological information differentiates different lexical semantics on the basis of different tonal categories (Gandour et al., 2000). Several neuroimaging studies have indicated that the acoustic information and the phonological information of lexical tones in native speakers are essentially two distinct auditory inputs, which exhibit different cognitive and physiological characteristics (Xi et al., 2010; Zhang et al., 2011, 2012; Yu et al., 2014, 2017). The phonological processing, which develops early in native speakers, is driven by an accumulation of perceptual development from the ambient native sound inputs (Kuhl, 2004). Thus, listeners’ speech processing performance can be modulated and reshaped by their experience with acoustic and phonological information (Chandrasekaran et al., 2009).

As for lexical tone perception, it has been reported that amusics perform worse than musically intact controls in their perception of non-native lexical tone categories (Nguyen et al., 2009; Tillmann et al., 2011; Wang and Peng, 2014), which reflects a domain-transferred acoustic processing deficit across the domains of music and speech. However, it remains unclear whether the native lexical tone perception deficits in tone language speakers with amusia (Nan et al., 2010; Jiang et al., 2012; Huang et al., 2015; Zhang et al., 2017a; Zhang and Shao, 2018) are merely due to the similar domain-transferred deficit in acoustic processing or additionally caused by reduced higher-level phonological processing. Answers to this question can help uncover whether and how reduced lower-level acoustic processing could negatively influence higher-level phonological processing. In order to fill the aforementioned research gap, the current P300 study examined the contrast between acoustic information and phonological information as the contrast between within-category and across-category variations by adopting a modified categorical perception (CP) paradigm (see Yu et al., 2014, 2017), which directly dissociates the acoustic and phonological processing of lexical tones for native amusic and control participants. According to model of Fujisaki and Kawashima (1971), lower-level acoustic processing is required to discriminate within-category discrimination pairs by the physical F0 difference, while higher-level phonological processing is mainly employed to conduct between-category discrimination tasks based on the information of phonemic categories. Moreover, the CP paradigm is more suitable because it reflects a fine-grained processing mode along a pitch continuum, with the perceptual pitch differences being relatively small.

To this end, the present P300 study compared Mandarin-speaking amusic and control participants’ behavioral and ERP responses to within-category and between-category deviants in both speech and nonspeech contexts. For native speakers, there appears to be some carry-over influence of the long-term phonological processing of lexical tone on the perception of the pitch counterparts from the speech to the nonspeech domain (Xu et al., 2006). Consequently, incorporating nonspeech stimuli enabled us to investigate whether the potential acoustic/phonological processing deficit in amusia is domain-specific or domain-general. All the stimuli were presented in an active oddball paradigm, randomly interspersing standards having the rising pitch contour of a high rising tone (Tone 2 in Mandarin) with equally spaced deviants of two types: a within-category deviant – an exemplar of the same linguistic category as the standard (Tone 2) but with a different pitch contour slope – and a between-category deviant – an exemplar of a distinct tone category (Tone 1 in Mandarin) from the standard, also with a different pitch contour slope. As consistently indicated in previous ERP studies of tone language speakers with amusia, the early-occurring components of P1, N1, and N2 were not significantly different between the amusic and control groups (Nan et al., 2016; Zhang and Shao, 2018), and the deficits occurred at the later attentive stage (P300 component) when consciously detecting lexical tone changes (Zhang and Shao, 2018). In this study, the amusic and control participants’ sensitivity index (*d'* score), reaction time (RT), mean amplitude, and peak latency of the P300 component were specifically analyzed to determine which types of deviant – within-category or between-category – in which context – speech or nonspeech – were different between the amusics and the controls.

In particular, we considered both behavioral and P300 evidence in favor of the following hypotheses: the amusics would show less sensitivity to a within-category deviant due to a domain-transferred inferior skill in acoustic processing. If the higher-level phonological processing – developed early in native speakers – is robust enough to resist the influence of reduced acoustic sensitivity, it is possible that the behavioral and P300 indices in responses to a between-category deviant would be similar between the two subject groups. Alternatively, if the acoustic processing deficits further extend to a reduced phonological processing of lexical tone categories, it is possible that Mandarin-speaking amusics would show compromised behavioral and neural processing of between-category variations compared to musically intact controls.

## Materials and Methods

### Participants

Fifteen amusic participants (seven females) and 15 musically intact control participants (seven females) from universities in Mainland China matched for chronological age, gender, and level of education participated in the current study. The sample size of the amusic and control participants in the current study was largely comparable to that usually reported in previous behavioral or ERP studies on lexical tone perception in amusics (cf. Jiang et al., 2012; Huang et al., 2015; Nan et al., 2016; Zhang et al., 2017a; Zhang and Shao, 2018). All the participants were right-handed and native Mandarin speakers from northern China with no reported history of hearing impairment, neurological illness, or long-term formal musical training. To identify the presence or absence of amusia, the online Montreal Battery of Evaluation of Amusia^1^ was utilized at the screening stage, with the cutoff average score set to 71% (Peretz et al., 2008). This online version includes two pitch-based subtests (out-of-key and mistuned subtests) and one rhythm/duration-based subtest (offbeat subtest), and it has been widely adopted as a diagnostic tool for tone language speakers with amusia (e.g., Wong et al., 2012; Wang and Peng, 2014; Zhang et al., 2017a; Zhang and Shao, 2018). The mean accuracy of the three subtests was 60.67% for the amusics and 83.73% for the controls. The results of an independent-samples *t*-test confirmed that both the global score and the scores of the three subtests for the amusic participants were much lower than those of the control participants (all *p*s < 0.001). The demographic characteristics of all the participants are presented and summarized in Table 1. Approval for this study was obtained from the Human Research Ethics Committee of the Shenzhen Institutes of Advanced Technology, the Chinese Academy of Sciences.

**Table 1:** Control and amusic participants’ sample size, chronological ages, and Online Identification Test of Congenital Amusia scores. The results (*p*) of independent-samples *t*-tests comparing the two groups are also reported. M, male; F, female; SD, standard deviation.

	Controls	Amusics	*p*
No. of participants	15 (8 M, 7 F)	15 (8 M, 7 F)	N/A
Age (SD)	22.87 years (2.85)	21.73 years (2.43)	*p* = 0.47
Online identification test of congenital amusia			
Out-of-key (SD)	88.67% (4.92%)	63.67% (11.30%)	*p* < 0.001
Offbeat (SD)	82.80% (7.73%)	62.00% (9.86%)	*p* < 0.001
Mistuned (SD)	78.73% (10.12%)	55.80% (10.94%)	*p* < 0.001
Mean score (SD)	83.73% (4.25%)	60.67% (5.49%)	*p* < 0.001

### Stimuli and Experimental Design

**Figure 1:**
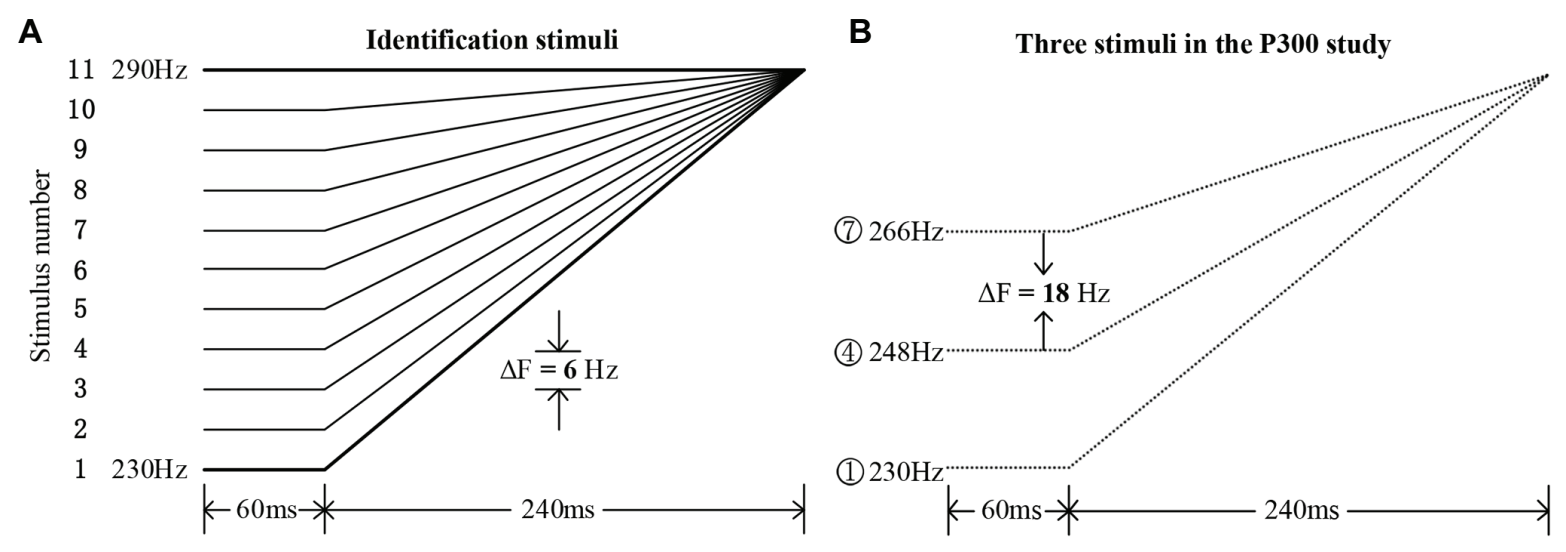
Experimental materials. **(A)** The 11 identification stimuli along a tone continuum ranging from typical Mandarin Tone 2 (stimulus #1) to Tone 1 (stimulus #11), with a step size of 6 Hz. **(B)** The three chosen stimuli in the rising set for the current P300 study (stimuli #1, #4, and #7), with an onset pitch difference of 18 Hz.

The Mandarin monosyllabic morpheme /i/ with the high-level tone (Tone 1, around 290 Hz) was recorded by a native female speaker (22,050 Hz sampling rate, 16-bit resolution). On the basis of the natural speech template with Mandarin Tone 1, a tone continuum containing 11 tone stimuli was resynthesized using the overlap-add re-synthesis function in Praat (Boersma and Weenink, 2009), with an equal step size of 6 Hz. The pitch contours are illustrated in Figure 1A; the procedures for synthesizing these stimuli followed those described in Peng et al. (2010). These pitch contours formed bilinear approximations (Wang, 1976) of high-rising (Tone 2, meaning “aunt”) and high-level (Tone 1, meaning “clothes”) tones in Mandarin. Eleven additional nonspeech counterparts were synthesized using the triangle wave (cf. Chen and Peng, 2016) with the same pitch contours as the speech stimuli. All the speech and nonspeech stimuli had a duration of 300 ms, with the pitch direction in the first 60 ms kept unchanged (see Figure 1). The nonspeech stimuli sound much lower perceptually when compared to the speech stimuli of the same intensity. This is because, compared with speech sound, the nonspeech sound (triangle wave) is much simpler in terms of spectro-temporal complexity. For the purpose of matching the loudness level, the speech and nonspeech stimuli were presented at 65 and 80 dB, respectively, which were based on the perceptual judgments of three native speakers (Zhang et al., 2013). Moreover, the intensity envelopes were closely matched and were kept constant across each speech and nonspeech continuum.

Based on a pilot identification test among several native Mandarin speakers who did not participate in the current P300 study, the obtained average boundary position (Finney, 1971) straddling the two categories was around stimulus #5 in both the speech and nonspeech continua. Then, in this P300 study, a rising set with three rising pitch contours, containing stimuli #1, #4, and #7 for both speech and nonspeech stimuli, was chosen (see Figure 1B). Based on the identification boundary, a within-category stimulus pair (#1 and #4) and a between-category stimulus pair (#4 and #7) were chosen for the ERP experiment using the oddball paradigm. Specifically, stimulus #4 was presented as the standard stimulus, stimulus #1 as the within-category deviant, and stimulus #7 as the between-category deviant. The onset acoustic distance between each type of deviant and standard was set to be 18 Hz, indicating that the between‐ or within-category deviants were equally spaced when compared with the standard stimuli. All stimuli were presented binaurally to both the control and amusic participants *via* a pair of E·A·R Tone 3A Insert Earphones manufactured by Etymotic Research Inc. Participants were seated in front of a monitor at a distance of approximately 60 cm in a sound-attenuated and electrically shielded chamber. All the sound stimuli were run by the E-Prime 2.0 program (Psychology Software Tools Inc., United States), which was also used to collect the behavioral RT and accuracy data.

Two oddball sessions (speech and nonspeech) were presented to each participant with a counterbalanced sequence. Each experimental session began with six standard trials, followed by 600 test trials in total, including 80% standards (480 trails), 10% within-category deviants (60 trials), and 10% between-category deviants (60 trials). The order of the test stimuli within each session was pseudo-randomized with at least three standard stimuli between any adjacent deviant stimuli. To fully control for the sequence effect, those three consecutive standard trials directly following deviants were disregarded in further ERP analyses. The stimulus-onset-asynchrony (SOA) jittered in the range from 1,100 to 1,500 ms. In each session, the stimuli were presented among five blocks, with 1-min breaks between blocks. Given that native speakers may find it harder to detect within-category deviants than between-category deviants (Zhang et al., 2012; Zheng et al., 2012), of the 15 participants in each subject group, eight were instructed to press the left button of the mouse after hearing the within-category deviants and the right button for the between-category deviants using their two thumbs, while the other seven were instructed to press the two buttons in the opposite manner. Moreover, all subjects were instructed not to press any mouse buttons after hearing the standards. The participants were asked to make their responses as fast as possible based on their first judgment. During the training stage, the three types of speech or nonspeech stimuli were played to each participant five, and only five, times and a practice run was presented before recording to familiarize participants with the task and stimuli. The participants were instructed to minimize their physical movements and eye blinks throughout the ERP experiment to reduce artifacts.

After the electrophysiological (EEG) recordings, all the participants carried out a behavioral identification posttest to the determine the identification boundary and further justify how they categorized the three chosen stimuli in this P300 study (i.e., stimuli #1, #4, and #7) along the speech or nonspeech continuum. For both the speech and nonspeech stimuli, each participant gave their responses by clicking the corresponding button on the keyboard labeled “Tone 1” or “Tone 2.” The 11 stimuli of each speech or nonspeech continuum (see Figure 1A) were repeated nine times in one block. Two blocks of tone identification, speech and nonspeech, were presented to each subject, with the order of the two blocks randomized.

### EEG Recording and Data Analyses

The EEG data were recorded in NetStation (V5.1.2) using an EGI (Electrical Geodesics, Inc.) GES 410 system with 64-channel HydroCel GSN electrode nets. Vertical and horizontal eye movements were monitored by electrodes placed on the supra‐ and infra-orbital ridges of each eye and electrodes near the external canthi of each eye, respectively. The continuous online recordings were referenced to the vertex electrode (Cz), digitized at a sampling rate of 1 kHz, and amplified with an analog band-pass filter of 0.1–75 Hz. Electrode impedances were generally kept below 20 kΩ, considerably below the 50 kΩ threshold recommended by the manufacturer (Electrical Geodesics, 2006).

For the analysis of the posttest behavioral data, the identification score was calculated as the percentage of responses in which the participants identified a particular sound as resembling either tone category (Tone 1 or Tone 2). The boundary position, defined as the corresponding 50% crossover point in a continuum, was obtained using Probit analysis (Finney, 1971). For the analysis of the behavioral data in this P300 study, both the sensitivity index (*d′* score) and mean RT of each participant’s behavioral responses were computed. For the analysis of the behavioral data in P300 paradigm, the sensitivity index *d′* (Macmillan and Creelman, 2008), was calculated as the *z*-score of the hit rate (corresponding button-press responses to deviant) minus that of the false alarm rate (any button-press responses to the standards). For instance, for one specific subject, the hit rate (*H*) of between-category or within-category deviants was calculated as the ratio of pressing the corresponding mouse button to the total number of each type of the deviant, and the false alarm rate (*F*) of standard stimuli was calculated as the ratio of pressing any mouse button to the total number of standard stimuli (since the participants were instructed not to press any button when the standard was presented). Then, the *d*′ score of between-category or within-category deviants was calculated using the following formula: *d′* = *z* (*H*) – *z* (*F*). Furthermore, the *d′* score of between-category deviants minus that of within-category deviants is referred to as the “peakedness *d′*” (Jiang et al., 2012). The RTs were analyzed for the correct responses to within‐ and between-category deviants, respectively, and those that fell outside of two SDs from the participant’s mean RT were not included.

**Figure 2:**
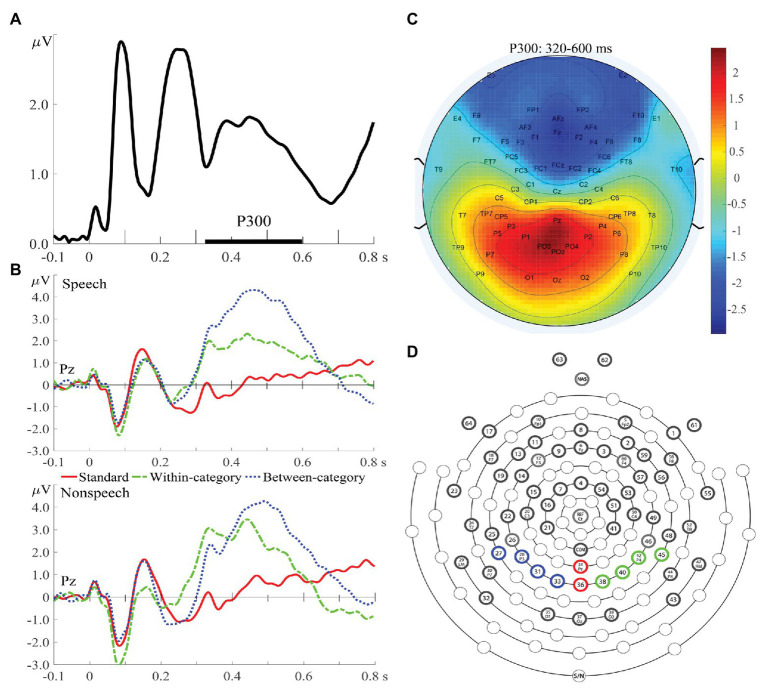
**(A)** Global field power averaged across all experimental conditions and across both groups of subjects. **(B)** Event-related potential (ERP) waves averaged from all subjects for speech (upper) and nonspeech (lower) contexts at Pz. **(C)** Topographic distribution map of P300 amplitudes (320–600 ms) averaged from standards and deviants. **(D)** A schematic view of the electrode array (64-channel HydroCel GSN), with four electrodes shaded with blue circles representing the region on the left hemisphere for the P300 component, two red electrodes in the midline, and four green electrodes on the right hemisphere.

For the offline analysis of the P300 data, the continuous EEG data were digitally refiltered with a 0.5–30 Hz band-pass filter and epoched with 100 ms of pre-stimulus intervals and 800 ms of post-stimulus intervals, with the pre-stimulus interval (−100–0 ms) used for baseline correction. Two electrodes attached to the left and right mastoids were used as offline re-references. Trials with ocular artifacts were excluded from averaging to ERP. Moreover, if more than 20% of the experimental trials for a particular participant were contaminated by ocular artifacts or body movements, the entire EEG data for that participant were excluded. Under this criterion, although we initially recruited 18 controls and 17 amusics to conduct this study, the EEG recordings obtained from five participants (three controls; two amusics) were excluded from further analyses due to excessive artifacts. The current study focused on the mean amplitude and peak latency of the P300 component at the later attentive stage. The time window of the P300 component (320–600 ms) was determined from global field power (Zheng et al., 2012; Zhang et al., 2013), calculated by taking the square root of the mean square ERP values averaged across all electrodes, all experimental conditions, and all subjects (Figure 2A), and further confirmed from the ERP waveforms (Figure 2B). The electrode locations of P300 were constrained to the region of interest on the basis of previous P300 studies and confirmed by the topographic distribution map (Figure 2C) in this study. In total, 10 parieto-occipital electrodes where the P300 amplitude was expected to peak (Polich, 2007) were chosen in terms of different brain hemispheres (Figure 2D): left (P1, P3, P5, and PO3), middle (Pz, POz), and right (P2, P4, P6, and PO4). Specifically, difference waves were obtained by subtracting the standard ERPs from each type of deviant ERPs and further submitted to statistical analyses. The amplitude of the P300 difference wave was determined by calculating the adaptive mean amplitude (Scerif et al., 2006) over electrodes within the specified time window (320–600 ms). The peak latency of the P300 difference wave was defined as the timing point corresponding to the maximal point of the second-order polynomial fitted curve to the difference wave.

## Results

### Posttest Identification Results

**Figure 3:**
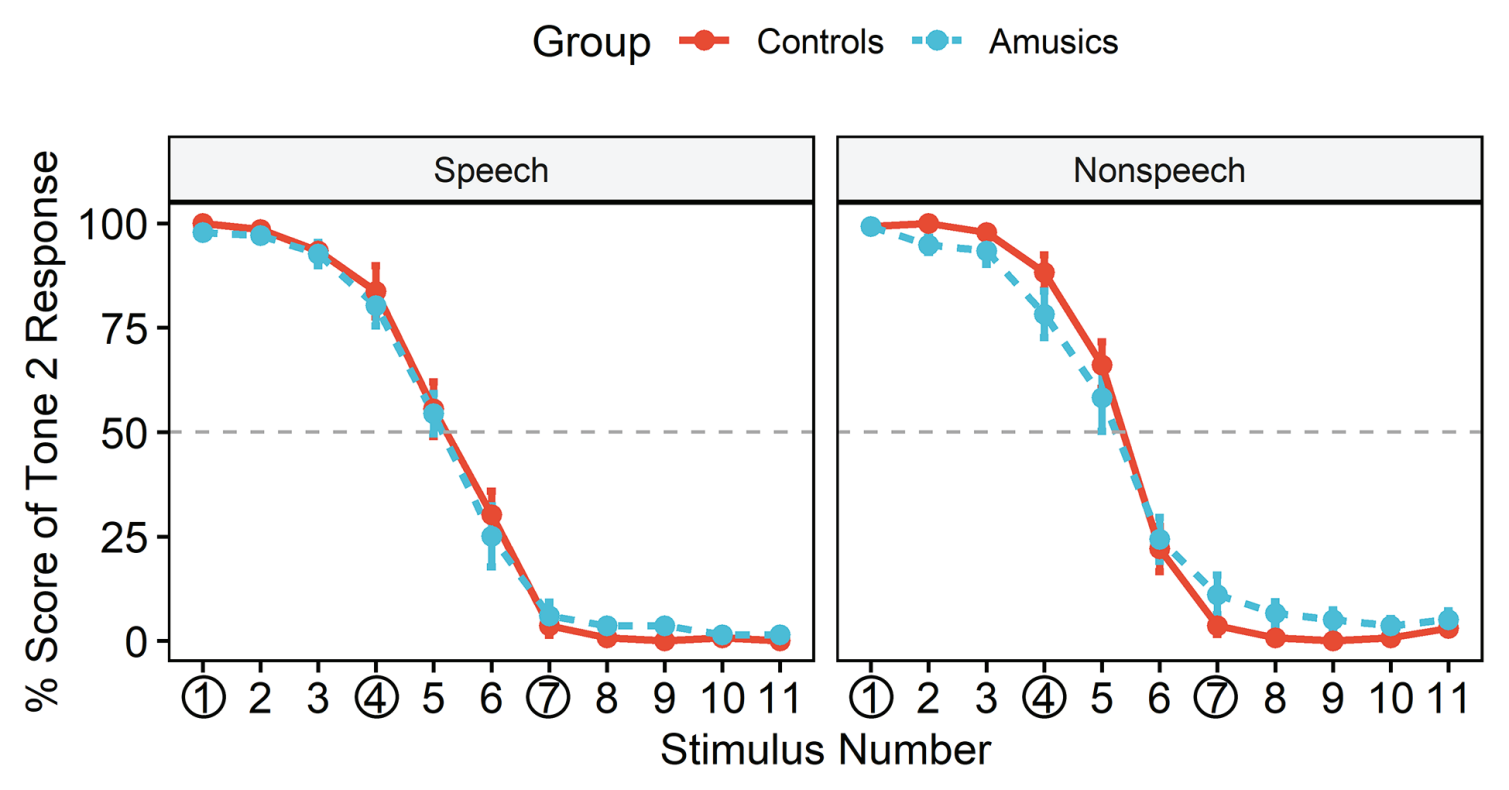
The identification curves of Tone 2 responses in the two groups, where the red solid lines represent responses in the control group and the blue dashed lines represent responses in the amusic group. Error bars: +/−1 standard error.

Figure 3 shows the posttest identification curves in the speech and nonspeech contexts among the controls and amusics. The typical stimuli at the rising end (stimulus #1) were consistently perceived as Mandarin Tone 2, with an identification rate close to 100% in both groups. All participants in most cases perceived stimulus #4 as belonging to the same category as stimulus #1 (i.e., “Tone 2”) for both the speech stimuli (Controls: 83.73%; Amusics: 80.27%) and the nonspeech stimuli (Controls: 88.27%; Amusics: 78.27%), while they perceived stimulus #7 as a different sound category (i.e., “Tone 1”) both for the speech stimuli (Controls: 96.33%; Amusics: 93.93%) and the nonspeech stimuli (Controls: 96.33%; Amusics: 88.93%).

The obtained mean boundary positions (SDs) were 5.14 (0.67) and 5.15 (0.59) in speech and 5.34 (0.57) and 5.31 (0.73) in nonspeech for the controls and the amusics, respectively. The boundary position was submitted to a two-way ANOVA with *group* (controls vs. amusics) as a between-subjects factor and *stimulus type* (speech vs. nonspeech) as a within-subjects factor. Greenhouse-Geisser corrections were conducted when appropriate. Neither the main effects nor the interaction effect reached significance (all *p*s > 0.05), indicating that the boundary position straddling the two categories was similar in the two subject groups and for different stimulus types. Therefore, the posttest identification results confirmed that both the control and amusic participants in this study indeed perceived #4 vs. #7 as a between-category contrast and #1 vs. #4 as a within-category contrast.

### Behavioral Results: *d*' Score and Reaction Time

**Figure 4:**
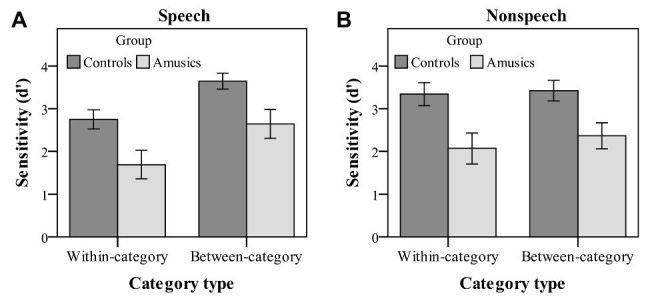
Sensitivity index (*d*' scores) of within-category and between-category deviants in speech **(A)** and nonspeech **(B)** stimuli for the control (dark bars) and amusic (light bars) participants. Error bars: +/−1 standard error.

Figure 4 displays both the control and amusic participants *d*′ scores for detecting within-category and between-category deviants in speech and nonspeech contexts. A three-way repeated measures ANOVA was conducted on the sensitivity index *d*′ by indicating *stimulus type* (speech vs. nonspeech) and *category type* (within-category vs. between-category deviant) as two within-subjects factors and *group* (controls vs. amusics) as a between-subjects factor. A significant main effect of *group*, *F*(1, 28) = 12.91, *p* = 0.001, *η_p_*^2^ = 0.32, was observed on *d′* scores, while none of its two-way and three-way interactions with other factors reached significance (all *p*s > 0.05), indicating that the amusics were overall less accurate than the controls in detecting both within-category and between-category deviants behaviorally for both the speech and nonspeech stimuli.

Moreover, there were a significant main effect of *category type* on *d*′ values [*F*(1, 28) = 26.56, *p* < 0.001, *η_p_*^2^ = 0.49] and a significant interaction of *category type* by *stimulus type* [*F*(1, 28) = 16.77, *p* < 0.001, *η_p_*^2^ = 0.38]. No other effects were significant. Next, a simple main effect analysis of the *category type* × *stimulus type* interaction was performed with Bonferroni adjustment. First, both groups of participants showed a much higher sensitivity to the between-category deviants (mean = 3.14) as compared with the within-category deviants (mean = 2.22) in the speech context [*F*(1, 28) = 39.20, *p* < 0.001, *η_p_*^2^ = 0.58], while the *d*′ values of the two types of deviants were not different from each other in the nonspeech context [*F*(1, 28) = 2.00, *p* = 0.17, *η_p_*^2^ = 0.07]. Then, a follow-up analysis of peakedness *d*′ (the *d*′ score of between-category deviants minus that of within-category deviants) was further conducted in the speech context, with no group differences being detected between the control and amusic groups [*t*(28) = −0.18, *p* = 0.86]. Second, the *d*′ value of between-category deviants in the speech stimuli was comparable to that in the nonspeech stimuli, *F*(1, 28) = 1.07, *p* = 0.31, *η_p_*^2^ = 0.04, while nonspeech (mean = 2.71) yielded a marginally higher *d′* value of within-category deviants than speech (mean = 2.22) for both the control and amusic groups, *F*(1, 28) = 3.72, *p* = 0.06, *η_p_*^2^ = 0.12.

**Figure 5:**
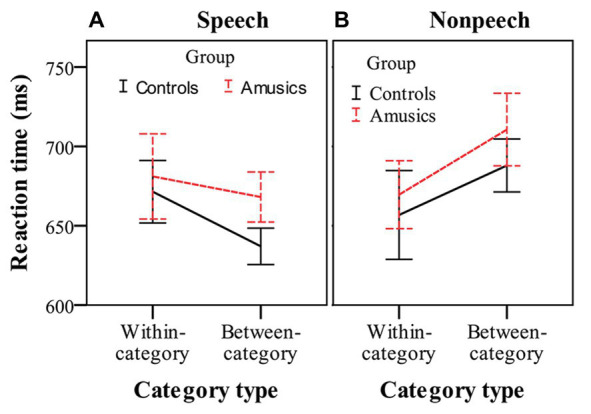
Reaction times in response to within-category and between-category deviants in speech **(A)** and nonspeech **(B)** contexts for the controls (black solid lines) and amusics (red dashed lines). Error bars: +/−1 standard error.

Figure 5 shows the RTs for detecting within-category and between-category deviants, in which participants made a behavioral response in speech and nonspeech contexts. RT was analyzed using three-way repeated measures ANOVA, with *group* as the between-subjects factor and *stimulus type* and *category type* as two within-subjects factors. The only significant effect was the interaction of *category type* by *stimulus type*, *F*(1, 28) = 13.53, *p* < 0.001, *η_p_*^2^ = 0.33. No other main effects or interactions reached significance (all *p*s > 0.05). These results indicated that the amusics and controls generally required similar amounts of RT to respond to the deviants. After this, simple main effect analyses were performed with Bonferroni adjustment. In the speech stimuli (see Figure 5A), the participants showed similar RTs to respond to within-category deviants (mean = 676.25 ms) and between-category deviants (mean = 652.55 ms), *F*(1, 28) = 2.40, *p* = 0.13, *η_p_*^2^ = 0.08. However, in the nonspeech stimuli (see Figure 5B), both groups of participants (controls and amusics) made a button press more quickly in response to the within-category deviants of stimulus #1 (mean = 663.17 ms) than in response to the between-category deviants of stimulus #7 (mean = 699.28 ms), *F*(1, 28) = 6.99, *p* < 0.05, *η_p_*^2^ = 0.20.

### Electrophysiological Results

**Figure 6:**
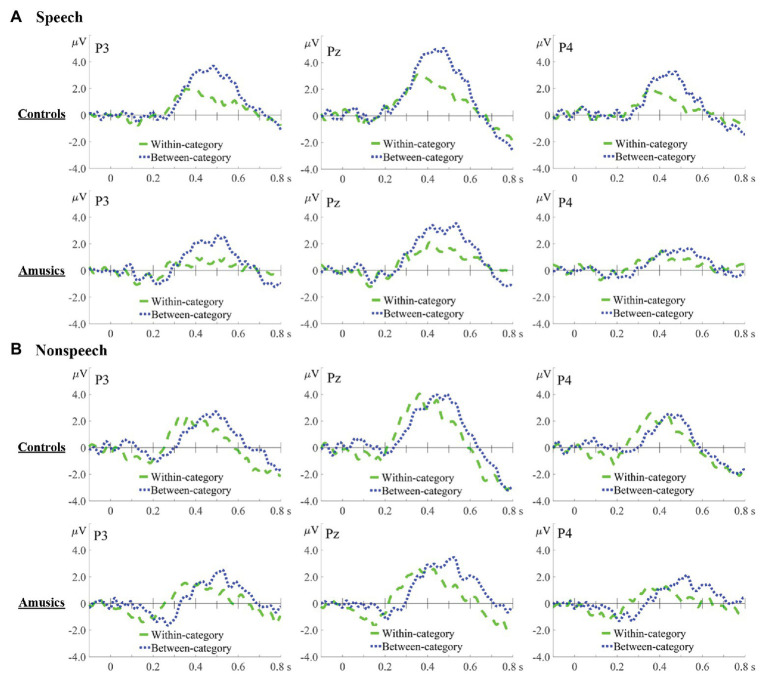
Difference waves in speech **(A)** and nonspeech **(B)** contexts for within-category deviants minus standards (green dashed lines) and between-category deviants minus standards (blue dotted lines) in the controls and amusics, which are displayed at three representative electrodes: P3 (left), Pz (middle), and P4 (right).

**Figure 7:**
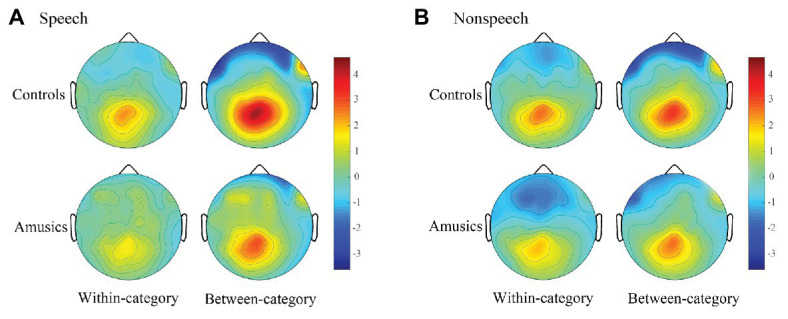
Topographic maps in speech **(A)** and nonspeech **(B)** contexts which demonstrate the scalp distributions of the P300 amplitudes (320–600 ms) of within-category and between-category difference waves (deviants minus standards) across the controls and amusics.

Difference waves were obtained by subtracting the standard ERPs from each of the deviant ERPs. Figures 6, 7, respectively, show the P300 difference waves of the within‐ and between-category deviants and their topographic voltage maps elicited from both the control and amusic participants in the speech and nonspeech stimuli. Four-way repeated measures ANOVAs were conducted on both the peak latency and mean amplitude of the P300 difference wave (320–600 ms), with three within-subjects factors – *stimulus type* (speech vs. nonspeech), *category type* (within-category vs. between-category deviant), and *hemisphere* (left, middle, and right) – and one between-subjects factor, *group* (controls vs. amusics). Corrections for violations of sphericity were made using the Greenhouse-Geisser method whenever necessary.

#### P300 Amplitude

**Figure 8:**
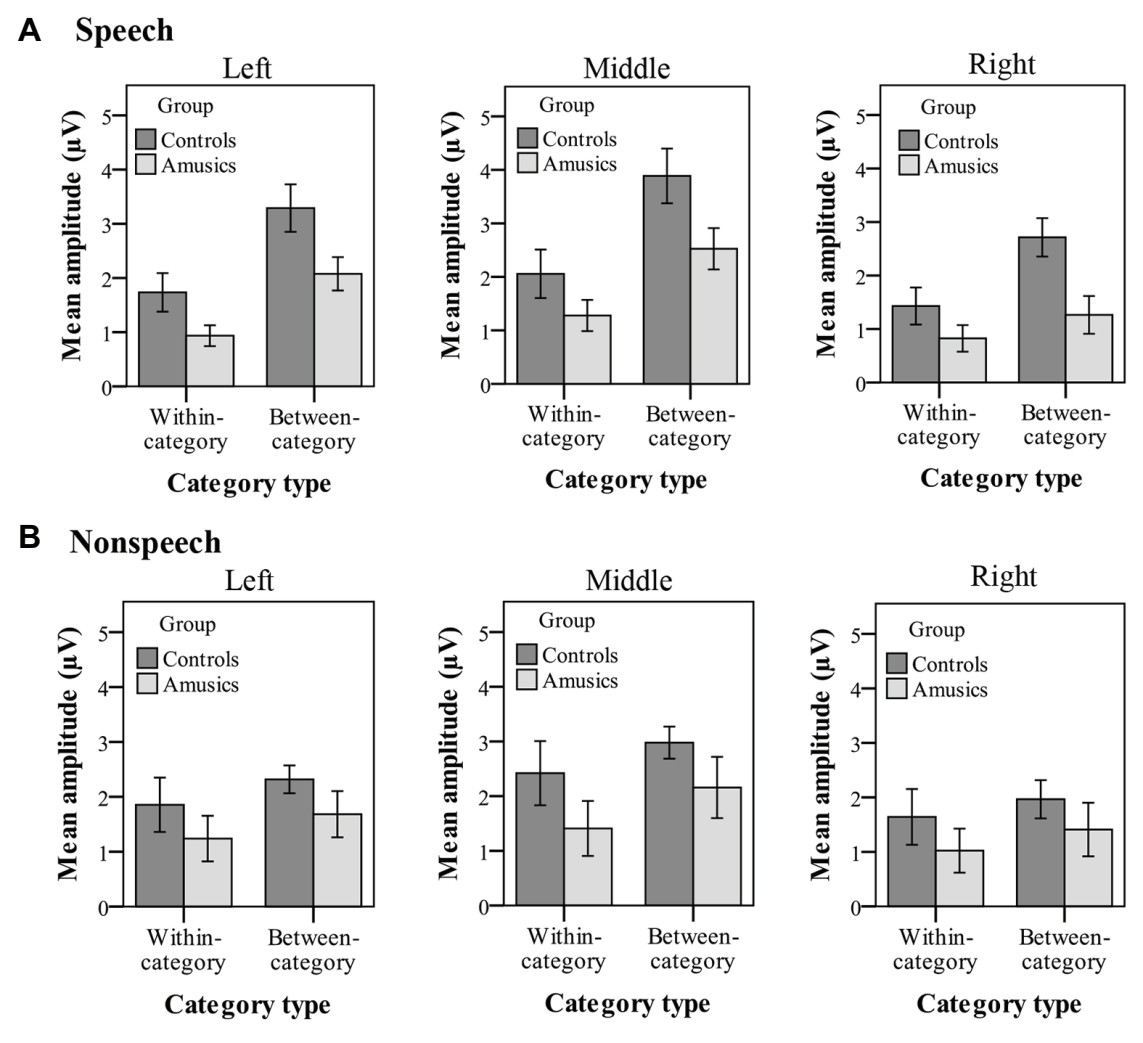
Mean P300 amplitude of within-category and between-category difference waves elicited in **(A)** speech stimuli and **(B)** nonspeech stimuli for both groups. The P300 amplitude was calculated from 320 to 600 ms by averaging the voltage measured at electrodes on the left hemisphere (P1, P3, P5, and PO3), in the midline (Pz, POz), and on the right hemisphere (P2, P4, P6, and PO4), respectively. Error bars: +/−1 standard error.

Figure 8 plots the mean P300 amplitudes of within-category and between-category difference waves among the two groups of subjects for both speech and nonspeech stimuli. With regard to the P300 amplitude, significant main effects of *group* [*F*(1, 28) = 8.26, *p* < 0.05, *η_p_*^2^ = 0.21], *category type* [*F*(1, 28) = 14.11, *p* = 0.001, *η_p_*^2^ = 0.34], and *hemisphere* [*F*(2, 56) = 29.28, *p* < 0.001, *η_p_*^2^ = 0.51] were observed. Moreover, there was a significant two-way interaction of *category type* × *hemisphere* [*F*(2, 56) = 10.31, *p* = 0.001, *η_p_*^2^ = 0.27] and a significant three-way interaction of *category type* × *hemisphere* × *stimulus type* [*F*(2, 56) = 4.02, *p* < 0.05, *η_p_*^2^ = 0.13]. Firstly, these results indicated that a greater P300 amplitude was elicited from the controls than from the amusics for the neural processing of both within‐ and between-category stimuli in both the speech and nonspeech stimuli (see Figure 8).

**Figure 9:**
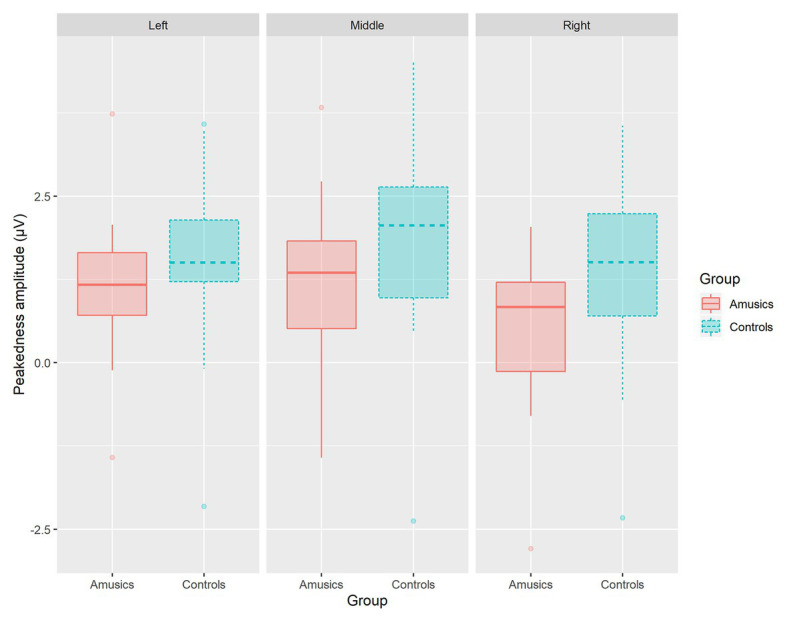
The boxplot of peakedness amplitude (the amplitude of between-category deviants minus that of within-category deviants) between the controls and amusics in the speech context.

Next, the *category type* × *hemisphere* × *stimulus type* interaction was further analyzed in terms of different stimulus types. First, in the speech context, there were significant main effects of *category type* [*F*(1, 28) = 26.08, *p* < 0.001, *η_p_*^2^ = 0.47] and *hemisphere* [*F*(2, 58) = 23.27, *p* < 0.001, *η_p_*^2^ = 0.45] and a significant interaction between *category type* and *hemisphere* [*F*(2, 58) = 13.01, *p* < 0.001, *η_p_*^2^ = 0.31]. Further analyses showed that in the speech stimuli, at all the electrodes on the midline and the left and right hemispheres, the processing of between-category deviants elicited a greater P300 amplitude than the processing of within-category deviants (all *p*s < 0.001) for both groups. Then, a follow-up analysis of peakedness amplitude (the amplitude of between-category deviants minus that of within-category deviants) was performed in the speech context (see Figure 9), and the results revealed no group differences between the controls and amusics in any of the three recording sites (all *p*s > 0.05). Furthermore, electrodes on the midline elicited a larger P300 amplitude than electrodes in the left and right hemispheres for the processing of both types of deviants in speech (all *p*s < 0.05). The left and right hemispheres were not significantly different from each other (*p* = 0.46) for the processing of within-category deviants in speech, while the P300 amplitude of the response to between-category deviants in the speech stimuli was larger over the left hemispheric recording sites (mean = 2.68 μV) than that in the right hemisphere (mean = 1.98 μV; *p* < 0.01).

Second, in the nonspeech context, only the main effect of *hemisphere* was detected, *F*(2, 58) = 19.61, *p* < 0.001, *η_p_*^2^ = 0.40. These results indicated that for all subjects, there was no significant difference in P300 amplitude for the processing of within-category and between-category deviants in nonspeech stimuli (see Figure 8B). Pair-wise comparison with Bonferroni adjustment showed that the P300 amplitude of the response to nonspeech stimuli at electrodes on the midline was significantly greater than that in the left and right hemispheres (all *p*s < 0.001), while the left and right hemispheres were not significantly different from each other (*p* = 0.11) for the neural processing of both types of deviants in nonspeech stimuli.

#### P300 Latency

For the peak latency of the P300 difference wave, there was a significant main effect of *category type* [*F*(1, 28) = 5.21, *p* < 0.05, *η_p_*^2^ = 0.16] and a significant four-way interaction of *category type* × *stimulus type* × *hemisphere* × *group* [*F*(2, 56) = 3.61, *p* < 0.05, *η_p_*^2^ = 0.11]. No other effects reached significance. Next, simple main effect analysis indicated that in the speech context, no effects reached significance (all *p*s > 0.05), indicating that in the speech stimuli, the peak latency elicited from within-category deviants was similar to that elicited from between-category deviants. In the nonspeech context, only the main effects of *category type* [*F*(1, 28) = 9.13, *p* < 0.01, *η_p_*^2^ = 0.25] and *hemisphere* [*F*(2, 56) = 3.86, *p* < 0.05, *η_p_*^2^ = 0.12] reached significance. The significant main effect of *category type* indicated that for both the controls and amusics, the mean peak latency of within-category deviants (mean = 424.22 ms) shifted earlier compared to that of between-category deviants (mean = 465.64 ms) in the nonspeech stimuli. Regarding *hemisphere*, however, pair-wise comparison with Bonferroni adjustment showed that none of the peak latencies between two hemispheres differed from the others (all *p*s > 0.05). Furthermore, these statistical results also implied that the group difference was not significant for the P300 latency in either the speech or nonspeech contexts.

## Discussion

### Reduced Attentive Processing of Between-Category Information of Lexical Tones in Mandarin-Speaking Amusics

In order to illuminate the level of lexical tone processing difficulties in Mandarin-speaking amusics, this study manipulated tonal category (within-category/across-category) along a pitch continuum to directly dissociate acoustic processing from the phonological processing of lexical tones for native speakers, an approach that was widely adopted in previous ERP studies (Xi et al., 2010; Zhang et al., 2012; Yu et al., 2014, 2017). A posttest identification test after the current P300 experiment confirmed that both the controls and amusics perceived (1) stimulus #1 (within-category deviant) as the same tone category as stimulus #4 (standard) and (2) the equally spaced stimulus #7 (between-category deviant) as a distinct tone category in both speech and nonspeech contexts (see Figure 3). As reflected by both the behavioral and electrophysiological measures, the *d*′ scores (see Figure 4) and P300 amplitudes (see Figure 8) in both speech and nonspeech contexts were systematically lower in the amusics than the controls when responding to both within‐ and between-category deviants, indicating that both lower-level acoustic processing and higher-level phonological processing were compromised among the tone language speakers with amusia, regardless of the linguistic status of the signal.

At the acoustic processing level, the result that the amusics exhibited reduced neural and behavioral processing of within-category deviants is in accordance with well-established findings that have consistently demonstrated that amusics are typically less sensitive to various fine-grained acoustic properties, such as the pitch information of music, nonspeech, and speech sounds (e.g., Peretz, 2001; Peretz et al., 2002; Nan et al., 2010; Jiang et al., 2012), and even to other acoustic correlates beyond pitch (Whiteford and Oxenham, 2017; Zhang et al., 2017a). Especially in regard to lexical tone perception, non-tonal language speakers with amusia demonstrate inferior performance of lexical tone discrimination in Mandarin Chinese (Nguyen et al., 2009; Tillmann et al., 2011) and Thai (Tillmann et al., 2011). Moreover, the native Mandarin (a tone language) speakers with amusia also show an impoverished perceptual skill in discriminating non-native Cantonese level tones (Wang and Peng, 2014). In brief, the aforementioned compromised processing of non-native lexical tones in amusics from different language backgrounds mainly reflects a domain-general acoustic processing deficit in the speech domain transferred from the music domain. Similarly, our findings further corroborated that the attentive processing of acoustic variations when detecting the within-category changes along native tonal stimuli is also compromised in both speech and nonspeech contexts. Thus, long-term tone language experience cannot compensate for the acoustic processing deficits in amusics even when the acoustic pitch is embedded in native tone categories, suggesting that the acoustic processing of pitch across different domains may share overlapping cognitive resources and/or processes (Patel, 2008, 2012).

At the phonological processing level, it has been proved that the developmental trajectory of native phonological categories starts early, from around 6 months in infants (Kuhl, 2004). The development of higher-level phonological perception in native speakers is thus driven by an accumulation of perceptual development from the tonal input of the ambient native sound environment (Chen et al., 2017). The main focus of this study was to figure out whether the higher-level phonological processing of native tone categories in amusics is more or less intact due to long-term exposure to the native tonal language or, alternatively, compromised at the attentive stage. The ERP component of P300 (P3b), which is associated with controlled processing in the attentive condition (Sutton et al., 1965), can act as an indicator of phonological processing capacity in the speech context (Maiste et al., 1995; Frenck-Mestre et al., 2005), and this feature may extend to nonspeech contexts (Chandrasekaran et al., 2009). The elicited P300 is modulated by the subject’s overall arousal level as resources are allocated to process different types of stimuli: the more demanding the task, the smaller the amplitude of the P300, and vice versa (Polich, 2007). In this study, the significantly greater amplitude elicited from the control group in response to between-category speech deviants implies the greater ease with which the controls engaged in phonological processing to distinguish between the F0 contours of distinct lexical tone categories. This difference also implies that the attentive phonological processing of lexical tone differences in Mandarin-speaking amusics might be compromised, which is in line with the reduced *d*′ values of between-category variations among the amusic cohort found in the behavioral measurements. To conclude, the results of the present experiment support the notion that the reduced acoustic processing in amusia is manifested broadly and profoundly and further persists into the higher-level phonological processing that involves the attentive processing of different lexical tone categories.

### The Influence of Lower-Level Acoustic Processing on the Higher-Level Phonological Processing of Lexical Tones

To accurately understand speech signals, listeners should process the basic acoustic characters and assign these highly variable speech sounds efficiently to various phonemic categories. These two types of information, acoustic information and phonological information are also dealt with when a native speaker processes lexical tones in Mandarin Chinese. Previous studies suggest that lower-level acoustic processing and higher-level phonological processing are represented differently in our human brain. One line of evidence reveals that speech is processed hierarchically along the auditory pathways, with the upstream areas (e.g., the dorsal STG areas) performing acoustic processing and the downstream regions (e.g., the ventral superior temporal sulcus, STS, and middle temporal gyrus, MTG regions) responsible for phonological processing (Wessinger et al., 2001; Okada et al., 2010; Zhang et al., 2011). The other line of evidence regarding brain lateralization indicates that although lexical tonal processing engages both hemispheres, pure acoustic processing tends to be processed in the right hemisphere, while phonological and semantic processing mainly occurs in the left hemisphere (Gandour et al., 2004; Gandour, 2006). Especially for Mandarin tone processing, Xi et al. (2010) found that between-category tonal deviants (i.e., phonological processing) elicited larger MMN than within-category tonal deviants (i.e., acoustic processing) in the left hemisphere, while the within-category deviants elicited larger MMN in the right hemisphere. At the attentive stage, Zhang et al. (2012) found that that for the P300 component, the amplitudes elicited by the within-category deviants were similar between the left and the right recording sites. However, the across-category deviants elicited greater P300 amplitudes in the left recording sites compared with the right sites. This hemispheric pattern found in Zhang et al. (2012) is the same as our current findings in both Mandarin-speaking controls and amusics when processing speech sounds, confirming that the phonological processing of lexical tones in tone language speakers happens in both hemispheres, although there is a bias toward the left side of our brain.

Although the aforementioned neuroimaging studies have implied that a dissociation exists between lower-level acoustic and higher-level phonological processing at different functional hierarchy levels (dorsal STG vs. ventral STS/MTG) and in different hemispheres (right vs. left), the way these two levels of processing interact with each other in lexical tone perception is not well understood. In one direction, phonological processing may influence acoustic processing. Zhang et al. (2011) found that compared to within-category variations, between-category stimuli elicit stronger activation in the middle MTG, reflecting the higher-level phonological representations. Meanwhile, activation on the dorsal plane of STG decreases significantly, suggesting that lower-level acoustic processing is modulated and dominated by higher-level phonological processing through “feedback mechanisms” (Hickok and Poeppel, 2007). Moreover, Zhao and Kuhl (2015) conducted a behavioral study by investigating the relative contribution of higher‐ and lower-level influences when both are present. They also concluded that higher-level linguistic categories dominate lower-level acoustics in lexical tone processing.

In the other direction, the “feed-forward mechanisms” (Binder, 2000; Scott and Wise, 2004) suggest that speech processing starts from the core auditory areas (STG) to downstream brain areas and then to more lateral and anterior regions, implying that initial bottom-up acoustic processing lays the foundation of phonological processing. The current findings provide direct evidence on the feed-forward mechanisms by showing that lower-level acoustics underlie higher-level phonological processing in lexical tone perception since the reduced acoustic processing skill in amusics extends to phonological processing in native perceivers. As congenital amusia is regarded as being inborn (Peretz, 2008), it is very likely that the impoverished acoustic pitch processing skill in this cohort interferes with the formation of native phonological categories from the very beginning. Taken together, the roles of both feed-forward and feedback mechanisms imply the existence of cortical dynamics (Zhang et al., 2011) for a bidirectional interaction between bottom-up acoustic analysis and top-down phonological processing in speech perception. The cortical dynamics support the dual-stream model of speech processing (Hickok and Poeppel, 2004, 2007) that claims reciprocal interactions across different brain regions. With more neuroimaging studies in the future, more will be revealed about the neurophysiological nature of the relative influence of acoustic and phonological processing in lexical tone perception.

### Neurophysiological Evidence for Preserved CP of Lexical Tones in Tone Language Speakers With Amusia

Categorical perception refers to a tendency for native listeners of a particular language to classify the sounds used in their language as discrete, categorical, and linguistic representations. The CP pattern of speech sounds is characterized by a sharp identification curve and an enhanced discrimination accuracy around the boundary position (Liberman et al., 1957). Thus, the cross-boundary benefit is likely to be the defining feature of CP (Massaro, 1987; Zhang, 2016). In other words, there is no support for claiming that CP occurs if there is no benefit for between-category discrimination compared to within-category discrimination. In terms of lexical tone perception, there is ample behavioral evidence that native perception of the Mandarin tonal continuum, due to the necessary involvement of contour tones, tends to be categorical, with higher sensitivity to between-category discrimination pairs relative to within-category pairs (e.g., Wang, 1976; Xu et al., 2006; Peng et al., 2010; Shen and Froud, 2016; Chen et al., 2017). Moreover, several ERP studies have provided electrophysiological evidence for the CP of lexical tones in both the pre-attentive stage (Xi et al., 2010; Yu et al., 2014, 2017; Shen and Froud, 2019) and the attentive stage (Zheng et al., 2012; Shen and Froud, 2019) by exhibiting more negative amplitudes (MMN) and more positive amplitudes (P300), respectively, elicited from between-category deviants relative to within-category deviants in native tone language speakers.

The previous studies investigating the CP of lexical tones in tone language speakers with amusia were all behavioral studies and drew different conclusions (Jiang et al., 2012; Huang et al., 2015; Zhang et al., 2017a). At the whole group level, Jiang et al. (2012) found an impaired CP of Mandarin tones in amusics, with no improvement for discrimination pairs that crossed the classification boundary. However, Zhang et al. (2017a) found that Cantonese-speaking amusics did perceive the lexical tones categorically, as indicated by the higher *d*′ for between-category pairs than for within-category pairs. Consistent with Zhang et al. (2017a), our behavioral results also indicate a higher sensitivity to between-category variations in Mandarin-speaking amusics. Besides, the current P300 results further offer electrophysiological evidence for claiming a preserved CP of lexical tones in Mandarin-speaking amusics. On the one hand, for both groups, controls and amusics, the across-category deviants did elicit a significantly higher amplitude than the within-category deviants in the speech context. On the other hand, the peakedness measure represents the magnitude of the benefit for discriminations that cross the identification boundary. In the current study, both the peakedness *d*′ of the behavioral index and the peakedness amplitude of the ERP index consistently showed no group differences.

The discrepancy regarding the CP pattern in the literature may largely be attributed to acoustic differences in the step size utilized in various studies. It is important to note that the step size of the discrimination pairs used in Jiang et al. (2012) was only 6 Hz, which falls in the range of just-noticeable differences (JNDs) for F0 discrimination (4–8 Hz) even among healthy Mandarin-speaking individuals (Liu, 2013). Given that the amusics generally showed a fine-grained pitch perception deficit, the 6-Hz acoustic difference used in the discrimination task might be too small to reveal the categorical nature in amusics. As indicated in Jiang et al. (2012), both the between‐ and within-category accuracies were close to the chance level due to a floor effect in the amusic group. The essential characteristic of the CP pattern reemerged in the tone language speakers with amusia when the acoustic distance of step size was enlarged (around 9–10 Hz on average in Zhang et al., 2017a, and 18 Hz in the current study). Furthermore, Huang et al. (2015) also confirmed that most Mandarin-speaking amusics (called “pure amusics” in their study) show a cross-boundary benefit when perceiving lexical tones behaviorally, in support of a preserved CP pattern, while a small portion of amusics (called “tone agnosics,” who showed very severe tone perception difficulties behaviorally, with an accuracy of 3 SDs below the mean of controls) still show a lack of CP of native tones. The view of subgroup differences in the CP of lexical tones in amusics needs to be corroborated with neurobiological investigation in future studies. At the very least, the present findings, from both behavioral and neural evidence, demonstrate a preserved CP pattern in Mandarin-speaking amusics at the whole group level.

Furthermore, as indicated by the indices of *d*′ value and P300 amplitude, the CP pattern of cross-boundary benefit in the speech context was not transferred to nonspeech counterparts for either group in this study, whereas such a cross-boundary benefit was often observed in the nonspeech context when it exhibited some of the critical features of speech (i.e., pitch; e.g., Xu et al., 2006; Xi et al., 2010; Zhang et al., 2017a), reflecting a carry-over influence of long-term phonological processing from the speech to nonspeech domain. In this study, for both the controls and amusics, the *d*′ values of between-category de*via*nts in speech were indeed comparable to those in nonspeech, whereas nonspeech yielded a higher *d*′ value of within-category deviants than speech (see Figure 4). As mentioned, CP is characterized as enhanced between-category discrimination as well as “dulled” within-category discrimination in the speech context, while within-category discriminations can be dulled less in the nonspeech context (Xu et al., 2006). The pattern that nonspeech yields higher within-category sensitivity than speech seems to be generalizable to all native tone-language speakers, be they controls (Xu et al., 2006; Zheng et al., 2012), musicians (Wu et al., 2015), or amusics (this study). Since the current study utilized a much larger step size (18 Hz) compared to other related studies (Xu et al., 2006; Zheng et al., 2012; Wu et al., 2015), this may lead to un-dulled within-category sensitivity in nonspeech that is comparable to the between-category sensitivity. Moreover, the within-category stimuli (#1) utilized in this study showed a much higher rising contour slope compared with the between-category stimuli (#7), which may partly explain the shorter RT (see Figure 5B) and earlier peak latency (see Figure 6B) in response to the within-category deviants in the nonspeech context. Nevertheless, no such differences between the two types of deviants were observed in the speech counterparts, probably due to the much easier detection of between-category speech deviants, which might make their processing time course shift earlier as well.

Another issue that arises with the current study is the underlying mechanisms to explain the reduced acoustic and phonological processing skills but preserved CP pattern in tone language speakers with amusia. This perceptual pattern in Mandarin-speaking amusics is essentially different from that found in non-tonal language speaking individuals who show both phonological processing deficit and an impaired CP in their perception of non-native lexical tones (e.g., Wang, 1976; Xu et al., 2006; Peng et al., 2010; Shen and Froud, 2016, 2019). These non-tonal language speakers show a phonological processing deficit due to a lack of exposure to lexical tones, but this could be improved with increased exposure and practice (Shen and Froud, 2016). However, for tone language speakers with amusia, although their phonological processing capacity was reduced to some extent, but not totally impaired due to the long-term native language exposure. According to the multistore model (Xu et al., 2006), three forms of memory – sensory memory and the short‐ and long-term forms of categorical memory – are involved in CP. Among these forms of memory, long-term categorical memory facilitates the categorization of speech sounds only in native speakers, and short-term categorical memory may become permanently preserved in long-term memory *via* long-term language exposure. Different from non-tonal language speakers, the presence of CP pattern in Mandarin-speaking amusics indicates that they might have normal or near normal long-term categorical memory (Jiang et al., 2012; Zhang et al., 2017a). The reduced acoustic and phonological processing skills in Mandarin amusics could possibly be attributed to difficulties in sensory memory and short-term categorical memory, respectively, which is in line with the proposed short-term pitch memory deficit in non-tonal language speakers with amusia (Gosselin et al., 2009; Tillmann et al., 2009, 2016; Albouy et al., 2015).

## Conclusion

In light of the positive transfer effects between music and language, it has been proposed that the music processing deficit in amusics would affect speech perception, such as lexical tone perception. This study aimed to uncover the neural mechanisms underpinning the lexical tone processing deficits in Mandarin-speaking amusics during the attentive stage (P300): whether such deficits in tone language speakers with amusia are merely caused by a domain-transferred deficit in lower-level acoustic processing or additionally influenced by reduced higher-level processing of phonological categories. Both the behavioral and P300 findings indicate that compared to the controls, both lower-level acoustic processing and higher-level phonological processing were reduced among the Mandarin-speaking amusics regardless of the linguistic status of the signal. Moreover, the current results imply an intact CP pattern in Mandarin-speaking amusics at the whole group level by exhibiting a cross-boundary perceptual benefit and a comparable peakedness in lexical tone perception, indicating that they may have normal or near normal long-term categorical memory due to long-term exposure to native tone categories. Considering the previous findings and the present results together, we suggest that long-term tone language experience may not compensate for the acoustic pitch processing deficit in tone language speakers with amusia but rather may be extended to the neural processing of the phonological information of lexical tones during the attentive stage.

## Data Availability Statement

The raw data supporting the conclusions of this article will be made available by the authors, without undue reservation.

## Ethics Statement

The studies involving human participants were reviewed and approved by Human Research Ethics Committee of the Shenzhen Institutes of Advanced Technology, the Chinese Academy of Sciences. The patients/participants provided their written informed consent to participate in this study.

## Author Contributions

FC and GP conceived and designed the experiment. FC implemented the experiment, collected, and analyzed the data. FC and GP interpreted the data and wrote the manuscript. All authors contributed to the article and approved the submitted version.

### Conflict of Interest

The authors declare that the research was conducted in the absence of any commercial or financial relationships that could be construed as a potential conflict of interest.

## Footnotes

^1^http://www.brams.org/amusia-public/
